# Transition to telemedicine and its impact on missed appointments in community-based clinics

**DOI:** 10.1080/07853890.2021.2019826

**Published:** 2021-12-31

**Authors:** Omolola E. Adepoju, Minji Chae, Winston Liaw, Tracy Angelocci, Paul Millard, Omar Matuk-Villazon

**Affiliations:** a Department of Health Systems and Population Health Sciences, College of Medicine, University of Houston, Houston, TX, USA; b Humana Integrated Health System Sciences Institute, University of Houston, Houston, TX, USA; c Lone Star Circle of Care, Georgetown, TX, USA

**Keywords:** COVID-19, telemedicine, patient no-shows, missed appointments, access to health care, federally qualified health centres

## Abstract

**Background and objective:**

The Coronavirus Aid, Relief, and Economic Security Act led to the rapid implementation of telemedicine across health care office settings. Whether this transition to telemedicine has any impact on missed appointments is yet to be determined. This study examined the relationship between telemedicine usage and missed appointments during the COVID-19 pandemic.

**Method:**

This retrospective study used appointment-level data from 55 Federally Qualified Health Centre clinics in Texas between March and November 2020. To account for the nested data structure of repeated appointments within each patient, a mixed-effects multivariable logistic regression model was used to examine associations between telemedicine use and missed appointments, adjusting for patient sociodemographic characteristics, geographic classification, past medical history, and clinic characteristics. The independent variable was having a telemedicine appointment, defined as an audiovisual consultation started and finalized via a telemedicine platform. The outcome of interest was having a missed appointment (yes/no) after a scheduled and confirmed medical appointment. Results from this initial model were stratified by appointment type (in-person vs. telemedicine).

**Results:**

The analytic sample included 278,171 appointments for 85,413 unique patients. The overall missed appointment rate was 18%, and 25% of all appointments were telemedicine appointments. Compared to in-person visits, telemedicine visits were less likely to result in a missed appointment (OR = 0.87, *p* < .001). Compared to Whites, Asians were less likely to have a missed appointment (OR = 0.82, *p* < .001) while African Americans, Hispanics, and American Indians were all significantly more likely to have missed appointments (OR = 1.61, *p* < .001; OR = 1.19, *p* = .01; OR = 1.22, *p* < .01, respectively). Those accessing mental health services (OR = 1.57 for in-person and 0.78 for telemedicine) and living in metropolitan areas (OR = 1.15 for in-person and 0.82 for telemedicine) were more likely to miss in-person appointments but less likely to miss telemedicine appointments. Patients with frequent medical visits or those living with chronic diseases were more likely to miss in-person appointments but less likely to miss telemedicine appointments.

**Conclusions:**

Telemedicine is strongly associated with fewer missed appointments. Although our findings suggest a residual lag in minority populations, specific patient populations, including those with frequent prior visits or chronic conditions, those seeking mental health services, and those living in metropolitan areas were less likely to miss telemedicine appointments than in-person visits. These findings highlight how telemedicine can enable effective and accessible care by reducing missed healthcare appointments.

**KEY MESSAGES**
- Telemedicine was associated with 13% lower odds of missed appointments.
- Patients with frequent medical visits or those living with chronic diseases were less likely to miss telemedicine appointments but more likely to miss in-person appointments.
- Patients seeking mental health services were less likely to miss telemedicine appointments but more likely to miss in-person appointments.
- Similarly, those living in metropolitan areas were less likely to miss telemedicine appointments but more likely to miss in-person appointments.

## Introduction

Health care spending in the United States (US) is the highest in the world, reaching $3.8 trillion in 2019, and accounting for nearly 18% of the nation’s gross domestic product [1]. Twenty-five cents out of every health care dollar is wasted though [2], and according to the National Academy of Medicine, one source of waste is operational inefficiencies such as missed appointments [3]. Patient no-shows, or missed appointments in health care, cost the US up to $50 billion annually [4]. In addition to causing loss in revenue, they also contribute to loss in productivity for clinics and under-utilization of clinic resources—while increasing wait times and delays for other patients to be seen by a medical provider. Repercussions of missed appointments go beyond clinic inefficiency. Patients with frequently missed appointments tend to experience poorer health outcomes and are less likely to utilize preventive health services [5,6]. Missed appointments are especially problematic for patients with chronic illnesses and mental health concerns. One study found that among those with chronic diseases, missed appointments serve as a harbinger for premature death [5].

Federally Qualified Health Centres (FQHCs) have matured into a principal part of the health system for thousands of medically underserved urban and rural communities that experience elevated poverty and health risks. They often serve populations in lower socioeconomic strata, racial and ethnic minority groups, and under- and uninsured patients, who may have worse access to care or perhaps higher rates of missed appointments. FQHCs qualify for federal funding under Section 330 of the Public Health Service Act that allows them to provide comprehensive primary care to all patients, regardless of their ability to pay. Nationally, nearly one in five Medicaid patients obtains care at an FQHC [7]. In Texas – a non-Medicaid expansion state – FQHCs face constant financial obstacles, including missed appointments that often impair operations [8].

The setting of the COVID-19 pandemic provided an opportunity to address telemedicine capacity through the Coronavirus Aid, Relief, and Economic Security (CARES) Act [9,10], which authorized FQHCs and other healthcare providers, to expand telemedicine services during the pandemic. Telemedicine provides an alternative to traditional face-to-face health care, offering a remote but cost-effective [11] means of communication between patients and providers. Over the past year, as technology use has become more integrated into our daily lives, more clinics have reported significant increases in telemedicine visits [12]. However, whether this transition to telemedicine has any impact on missed appointments is yet to be determined.

Missed appointments have previously been shown to be associated with a variety of factors, including a lack of sense of urgency to receive care, scheduling policy [8], fear and anxiety surrounding appointments, language barriers, forgetfulness, transportation-related issues, concern over service cost, weather [13], insurance coverage, and long lead times to appointments [8]. In primary care, the most common reasons for missing appointments are forgetfulness and miscommunication with clinic staff [14]. While these earlier studies have formed a great foundation in the effort to reduce inefficiencies associated with missed appointments, few [15,16] have examined the impact of telemedicine use on missed appointments. Investigating this role of telemedicine is particularly noteworthy because of its suggested potential in reducing missed appointments. Further, it is imperative to understand factors associated with missed appointments in order to design specific interventions for populations that may face greater odds of missing health care-related appointments. The goal of this study was to examine the relationship between telemedicine and missed appointments in clinics primarily designed to provide care to underserved populations between March and November 2020.

## Method

### Data

Data from the electronic medical records (EMRs) at a large FQHC network was used to identify the study population. This FQHC has 55 individual clinics across 6 counties in Texas. In 2019 alone, these clinics held 357,000 encounters with 93,000 unique patients. For the purposes of this study, administrative data on all patients who had one or more appointments were pulled over a period of 9 months (from 1 March to 30 November 2020). These dates reflect the initiation and ongoing pattern of telemedicine appointments in response to the COVID-19 pandemic.

Data included patient demographic information, appointment information, and encounter-level information on in-person and virtual visits. Appointments included telephone (1%), telemedicine (24%), and face-to-face visits (75%). For the purposes of this analysis, we combined telephone and telemedicine visits into one group. (In the first weeks of the pandemic before the launch of the telemedicine platform, telephone visits were used for patient management.)

### Measurement

The outcome of interest was having a missed appointment (no [0], yes [1]) after a scheduled and confirmed medical appointment. “Missed appointment” was defined by the clinical operations team as a patient who did not show up at all for the appointment OR who cancelled the appointment fewer than 2 h before the appointment time. The independent variable of interest was telemedicine appointment (no [0], yes [1]). “Telemedicine” was defined as an audiovisual consultation started and finalized via a telemedicine platform with which the FQHC contracted.

Other independent variables included patient sociodemographic variables (i.e. age, ethnicity, race, insurance coverage type), patient geographic classifications (i.e. distance in miles to clinic, metropolitan/nonmetropolitan status, residence in a medically underserved area [MUA] as defined by the Health Resources & Services Administration [HRSA]), medical appointment information [type and date of appointment]), and clinic characteristics (i.e. service lines offered, e.g. family practice, mental health, obstetrics/gynecology, paediatrics, senior care). Because the data provided did not include patient symptoms and/or medical diagnoses (factors that may affect whether the visit can occur via telemedicine), the research team used past-visit volume over the preceding 15 months as a proxy. Using this analogy, patients with 1–2 visits in 15 months before March 2020 were considered relatively healthy, those with 3–4 visits were considered to probably have acute exacerbations and/or chronic conditions necessitating follow-up, and those with 5+ visits were considered likely to have chronic conditions for which they frequently visit a clinic.

### Analysis

Descriptive analyses employing frequencies, proportions, means, and standard deviations were used to describe patient demographic characteristics. Chi-square tests were used to assess the strength of the relationship between each independent variable and missed appointments. Variables that were significant at the bivariate stage were included in the multivariate analysis. Because the unit of analysis for our multivariate modelling was at the appointment level, a mixed-effects logistic regression model assessed the relationship between missed appointments and telemedicine use, adjusting for patient sociodemographic characteristics, geographic classification, medical appointment information, and clinic characteristics. This allowed the investigators to account for the nested data structure and for repeated appointments of individual patients. Patient-assigned identification numbers were included in the model as a random effect. Fixed effects included all the independent variables mentioned earlier. Results from this initial model were stratified by appointment type (in-person vs. telemedicine) to understand whether certain types of patients were more/less likely to miss appointments if they have telemedicine vs. in-person visit. Sensitivity analyses were also conducted to test for changes with and without the inclusion of telephone appointments. This study was approved by an independent institutional review board in October 2020. All data management and analyses were performed using Stata 16.1. All statistical tests were two-sided, and findings were considered statistically significant at *p* < .05.

## Results

### Sample characteristics

Overall, the sample contained 278,171 appointments for 85,431 unique patients. The average count of medical appointments was 3.4 over the 9-month period. The sample comprised 39.2% males, 51.6% Hispanics, and 38.5% racial minorities (Table 1). Racial minority subgroups included Black or African American (11.9%), Asian (3.0%), and other races (23.6%). Children aged 0–18 years represented 46.4% of the sample, and older adults aged 65+ years represented 4.8%, while 48.9% were between 18 and 64 years of age. Regarding patient case-mix, 16.5% had private health insurance, 44.3% had Medicaid, 5.6% had Medicare, and 33.7% were uninsured. As for patient location, 96.1% of the analytic sample resided in metropolitan areas while 49.7% resided in MUAs. Of all patients, 40.1% had 1–2 visits in the 15 months preceding March 2020, 19% had 3–4 visits, and 40.9% had 5+ visits. The average patient distance from the clinic was 13.9 miles (SD = 21.4). During the study period (March to November 2020), 25% of all appointments were telemedicine appointments. The overall missed appointment rate was 18%.

### Bivariate analysis

Table 2 shows the bivariate associations of baseline characteristics by missed appointment status. Adults aged 18–64 were more likely to miss appointments compared to children and older adults (18.9% vs.17.0% vs. 13.9%, *p* < .001). Hispanic patients were more likely to miss appointments compared to non-Hispanic patients (18.6% vs. 17.2%, *p* < .001). African Americans reported the highest missed appointment rate (22.1%), while Asian patients reported the lowest missed appointment rate (12.7%). Those who had no insurance were more likely to miss appointments (20.2%) compared to Medicare enrollees (16.0%), Medicaid enrollees (17.6%), and privately insured individuals (11.8%) (*p* < .001). With respect to service lines, family practice had more missed appointments compared to mental health, obstetrics/gynecology, paediatrics, and senior care (20.2% vs. 16.5% vs. 16.9% vs. 17.3% vs 13.1%, *p* < .001). Those with telemedicine appointments were less likely to miss appointments compared to in-person/face-to-face visits (15.8% vs. 18.7%, *p* < .001).

**Table 1.** Unique patient baseline characteristics (*n* = 85,431).

Variable	Total, *n* (%)
Age	
<18	39,821 (46.6)
18–64	41,511 (48.6)
>64	4081 (4.8)
	Mean (SD)
Age at appointment	27.1 (20.5)
Gender	
Female	51,906 (60.8))
Male	33,507 (39.2)
Ethnicity	
Hispanic	44,101 (51.6)
Non-Hispanic	41,312 (48.4)
Race	
White	52,571 (61.6)
Black or African American	10,168 (11.9)
Asian	2540 (3.0)
American Indian, Alaska Native, other Pacific Is.	978 (1.2)
Mixed race	1744 (2.0)
Other	17,412 (20.4)
Metropolitan status of patient residence	
Nonmetropolitan	3340 (3.9)
Metropolitan	82,073 (96.1)
MUA status of patient residence	
Non-MUA	43,170 (50.3)
MUA	42,694 (49.7)
Visit history (December 2018 to February 2020)	
1–2 visits	34,224 (40.1)
3–4 visits	16,267 (19.0)
5+ visits	34,922 (40.9)

**Table 2.** Bivariate associations of baseline characteristics by missed appointment status (*n* = 278,171).

Variable	Total, *n* (%)	Missed appointment status		*p*-Value
		Made appointment (*n* = 228,308), *n* (%)	Missed appointment (*n* = 49,863), *n* (%)	
Age				<.001
<18	112,711 (40.5)	93,572 (83.0)	19,139 (17.0)	
18–64	153,110 (55.0)	124,105 (81.1)	29,005 (18.9)	
>64	12,350 (4.4)	10,631 (86.1)	1719 (13.9)	
Ethnicity				<.001
Non-Hispanic	139,134 (50.0)	115,154 (82.8)	23,980 (17.2)	
Hispanic	139,037 (50.0)	113,154 (81.4)	25,883 (18.6)	
Race				<.001
White	174568 (62.8)	144,795 (82.9)	29,773 (17.1)	
Black or African American	34,538 (12.4)	26,901 (77.9)	7637 (22.1)	
Asian	7104 (2.6)	6201 (87.3)	903 (12.7)	
American Indian, Alaska Native, other Pacific Is.	3202 (1.2)	2589 (80.9)	613 (19.1)	
Mixed race	6136 (2.2)	5034 (82.0)	1102 (18.0)	
Other	52,623 (18.9)	42,788 (81.3)	9835 (18.7)	
Insurance coverage				<.001
Private insurance	47,390 (17.0)	41,795 (88.2)	5595 (11.8)	
Medicare	16,234 (5.8)	13,638 (84.0)	2596 (16.0)	
Medicaid	123,868 (44.5)	102,108 (82.4)	21,760 (17.6)	
Uninsured	88,601 (31.9)	70,736 (79.8)	17,865 (20.2)	
Service line				<.001
Family practice	88,044 (31.7)	70,234 (79.8)	17,810 (20.2)	
Mental health	59,074 (21.2)	49,328 (83.5)	9746 (16.5)	
Obstetrics/gynecology	40,035 (14.4)	33,257 (83.1)	6778 (16.9)	
Paediatrics	84,671 (30.4)	69,981 (82.7)	14,690 (17.3)	
Senior care	6329 (2.3)	5503 (86.9)	826 (13.1)	
Metropolitan status				.01
Nonmetropolitan	9418 (3.4)	7829 (83.1)	1589 (16.9)	
Metropolitan	268,610 (96.6)	220,355 (82.0)	48,255 (18.0)	
Distance from clinic				.05
<5 miles	76,606 (27.5)	62,785 (82.0)	13,821 (18.0)	
5–10 miles	72,930 (26.2)	59,952 (82.2)	12,978 (17.8)	
10–20 miles	77,670 (27.9)	63,845 (82.2)	13,825 (17.8)	
20–50 miles	44,565 (16.0)	36,417 (81.7)	8148 (18.3)	
≥50 miles	6400 (2.3)	5309 (83.0)	1091 (17.0)	
MUA status				<.001
Non-MUA	142,496 (51.2)	115,854 (81.3)	26,642 (18.7)	
MUA	135,532 (48.7)	112,330 (82.9)	23,202 (17.1)	
Appointment type				<.001
Face-to-face appointments	207,621 (74.6)	168,871 (81.3)	38,750 (18.7)	
Telemedicine appointments	70,550 (25.4)	59,437 (84.2)	11,113 (15.8)	

*Note*. Missing data not included in statistical analyses.

Patient geographic residence was also associated with missed appointments. Those living in nonmetropolitan areas were less likely to miss appointments (16.9% vs. 18.0%, *p* = .01). While absolute differences were small (<0.5%), missed appointment rates were marginally higher for some distances (*p* = .05). Living in an MUA was associated with a lower missed appointment rate (17.1% vs. 18.7%, *p* < .001).

Table 3 displays the trends of telemedicine use and missed appointments over the course of the 9-month study period. Telemedicine use fluctuated between March and November 2020, initially at 4% of all appointments in March, peaking in April at 35% of all appointments, and slowly declining to 30% in June, 27% in September, and finally 24% in November. Missed appointment rates also changed over this time period. Missed appointment rates were highest at the beginning of the study in March 2020 at 19%, decreased to 18% in April, held relatively steady through September, and ultimately landed at 16% in November.

**Table 3.** Telemedicine use and missed appointments over time (1 March to 30 November 2020).

Month	Total appointments, *n*	Telemedicine appointments [*n* (%) of total appointments]	Missed appointments [*n* (%) of total appointments]
March 2020	34,164	1316 (3.9)	6606 (19.3)
April 2020	24,347	8496 (34.9)	4321 (17.7)
May 2020	25,407	8556 (33.7)	4437 (17.5)
June 2020	31,985	9445 (29.5)	5869 (18.3)
July 2020	33,255	10,144 (30.5)	6023 (18.1)
August 2020	31,948	8463 (26.5)	5687 (17.8)
September 2020	32,790	8697 (26.5)	5973 (18.2)
October 2020	33,788	7987 (23.6)	5854 (17.3)
November 2020	30,487	7446 (24.4)	5093 (16.7)

### Mixed-effects regression models

Results from both mixed-effects regression models are presented in Table 4. Adjusting for patient sociodemographic characteristics, geographic classification, visit history, and clinic characteristics, telemedicine appointments were associated with 13% lower odds of a missed appointment (OR = 0.87, *p* < .001). Compared to Whites, Asians were less likely to miss appointments (OR = 0.82, *p* < .001), while African Americans, persons of two or more races, and American Indians, Alaska Natives, and other Pacific Islanders were all significantly more likely to miss appointments (OR = 1.61, *p* < .001; OR = 1.19, *p* = .01; OR = 1.22, *p* < .01, respectively). Hispanics were also more likely to miss appointments (OR = 1.19, *p* < .001). Compared to working adults, children were less likely to miss appointments (OR = 0.83, *p* < .001), as were older adults (OR = 0.71, *p* < .001).

**Table 4.** Mixed-effect logistic regression model of the relationship between telemedicine and missed appointments.

Variable	OR	MV-adjusted OR^a^, 95% CI	*p*-Value
Appointment type			
Face-to-face appointment	Ref.		
Telemedicine appointment	0.87	0.84–0.89	<.001
Age			
18–64	Ref.		
<18	0.83	0.79–0.88	<.001
>64	0.71	0.65–0.77	<.001
Gender			
Male	Ref.		
Female	1.01	0.98–1.03	.64
Ethnicity			
Non-Hispanic	Ref.		
Hispanic	1.19	1.16–1.23	<.001
Race			
White	Ref.		
Black or African American	1.61	1.54–1.68	<.001
Asian	0.82	0.74–0.90	<.001
American Indian, Alaska Native, other Pacific Is.	1.19	1.05–1.35	.013
Mixed race	1.22	1.13–1.36	<.001
Insurance coverage			
Private insurance	Ref.		
Medicare	1.62	1.50–1.75	<.001
Medicaid	1.75	1.68–1.83	<.001
Uninsured	1.90	1.82–1.92	<.001
Service line			
Family practice	Ref		
Mental health	1.20	1.15–1.25	<.001
Obstetrics/gynecology	0.88	0.84–0.92	<.001
Paediatrics	0.94	0.89–1.00	.05
Senior care	0.74	0.67–0.81	<.001
Metropolitan status			
Nonmetropolitan	Ref.		
Metropolitan	1.06	0.98–1.14	.15
Distance from clinic			
<5 miles	Ref.		
5–10 miles	1.02	0.93–1.13	.59
10–20 miles	1.01	0.89–1.08	.71
20–50 miles	1.02	0.89–1.09	.80
≥50 miles	1.00	0.91–1.11	.93
MUA status			
Non-MUA	Ref.		
MUA	0.95	0.93–0.98	.001
Visit history (December 2018 to February 2020)			
1–2 visits	Ref.		
3–4 visits	1.67	1.61–1.74	<.001
5+ visits	1.61	1.56–1.66	<.001
Intraclass correlation coefficient for random effect			
	ICC	95% CI	Std. Err.
Patient	0.20	0.19–0.21	0.003

^a^ MV-adjusted OR, multivariate adjusted odds ratio.

^a^MV-adjusted OR = multivariate adjusted odds ratio.

Missed appointments also varied by insurance type. Compared to patients with private health insurance, patients with Medicaid (OR = 1.75; *p* < .001) or Medicare (OR = 1.62; *p* < .001) and those who were uninsured (OR = 1.90; *p* < .001) were all significantly more likely to have missed appointments. While residence in a metropolitan area was not significantly associated with missed appointments, patients residing in MUAs were significantly less likely to miss appointments (OR = 0.95; *p* = .001). Persons residing farther than 5 miles from the clinic were more likely to miss appointments compared to patients residing within 5 miles, though these differences were not statistically significant (OR = 1.02, *p* = .93; OR = 1.01, *p* = .89; OR = 1.02, *p* = .89; OR = 1.02; *p* = .91, respectively).

Compared to family practice clinics, the odds of missed appointments were higher in mental health (OR = 1.20, *p* < .001) but lower in pediatrics (OR = 0.94; *p* = .05), senior care (OR = 0.74, *p* < .001), and obstetrics/gynecology (OR = 0.88, *p* < .001). Results from the sensitivity analyses were largely consistent. When compared to patients with 1–2 visits in the prior 15 months, those with 3–4 past visits (OR = 1.67, *p* < .001) and those with 5+ past visits (OR = 1.61, *p* < .001) were significantly more likely to miss appointments. Conditional on the fixed-effects covariates, patient random effect composed approximately 20% of the total residual variance.

Table 5 shows missed appointments stratified by appointment type. Similar patterns were observed for in-person and telemedicine appointments across the following independent variables: age, race, ethnicity, and insurance coverage. However, other independent variables showed opposite trends. Compared to those accessing family practice services, those accessing mental health services were more likely to miss in-person appointments but less likely to miss telemedicine appointments. The same was true for the rural-urban divide, such that those living in urban areas were more likely to miss in-person appointments but less likely to miss telemedicine appointments. Compared to those with only 1–2 visits in the prior 15 months, those with 3–4 past visits and those with 5+ past visits were more likely to miss in-person appointments but less likely to miss telemedicine appointments. Patient random effects composed approximately 22% of the total residual variance for in-person appointments and approximately 26% of the total residual variance for telemedicine appointments.

**Table 5.** Mixed-effect logistic regression model of missed appointments, stratified by appointment type.

Variable	In-person	Telemedicine
Age		
18–64	Ref.	Ref.
<18	0.86*	0.68*
>64	0.65*	0.84*
Gender		
Male	Ref.	Ref.
Female	0.97	1.15*
Ethnicity		
Non-Hispanic	Ref.	Ref.
Hispanic	1.13*	1.46*
Race		
White	Ref.	Ref.
Black or African American	1.63*	1.53*
Asian	0.80*	0.86
American Indian, Alaska Native, other Pacific Is.	1.16*	1.28
Mixed race	1.31*	0.99
Insurance coverage		
Private insurance	Ref.	Ref.
Medicare	1.63*	1.75*
Medicaid	1.80*	1.69*
Uninsured	1.95*	1.75*
Service line		
Family practice	Ref.	Ref.
Mental health	1.57*	0.78*
Obstetrics/gynecology	0.90*	0.92
Paediatrics	0.95	0.97
Senior care	0.83*	0.53*
Metropolitan status		
Nonmetropolitan	Ref.	Ref.
Metropolitan	1.15*	0.82*
Distance from clinic		
<5 miles	Ref.	Ref.
5–10 miles	0.97	1.15
10–20 miles	0.94	1.07
20–50 miles	0.95	1.11
≥50 miles	0.95	1.22*
MUA status		
Non-MUA	Ref.	Ref.
MUA	0.95*	0.94
Visit History (December 2018 to February 2020)		
1–2 visits	Ref.	Ref.
3–4 visits	1.88*	0.91*
5+ visits	1.82*	0.92*
Intraclass correlation coefficient for random effect		
	ICC (Std. Err.)	ICC (Std. Err.)
Patient	0.22 (0.004)	0.26 (0.009)

*p < .05.

Supplementary tables show the pattern of missed appointments in patients 0–18 years and adults 19+. In patients 0–18 years, telemedicine was strongly associated with a lower likelihood of missed appointments (OR: 0.76, *p* < .001). This result is similar to the finding in the overall population model where telemedicine also exhibited a lower likelihood of missed appointments (OR: 0.83). The control variables (gender, race, ethnicity, insurance coverage, metropolitan residence, visit history), exhibited similar associations with missed appointments (as observed in the overall population model), with the exception of distance to clinic and mental health service line. Unlike the overall population model that showed an insignificant dose-response relationship with missed appointments, distance to clinic exhibited a significant dose-response relationship for 5–10 miles and 10–20 miles away from the clinic (i.e. the further to the clinic, the higher the likelihood of a missed appointment). Mental health service was not associated with missed appointments in patients 0–18 years, however, in the overall model, the mental health service line was significantly associated with missed appointments. In adult patients, the results were similar to the results from the overall population model. There were no differences in the direction or significance of the association.

## Discussion

This study examined associations between telemedicine visits and missed appointments in clinics primarily designed to provide care to underserved populations. To the best of our knowledge, this is one of the few multi-clinic studies to examine the impact of telemedicine on missed appointments in community-based clinics during the COVID-19 pandemic. We found that compared to in-person visits, the odds of missing a telemedicine appointment were significantly lower. Living in an MUA and being of Asian descent were also associated with lower rates of missed appointments. In contrast, individuals who were African American, Hispanic, uninsured, or seen for a family medicine appointment had higher rates of missed appointments. These findings align with earlier reports of reduced missed appointments following the transition to telemedicine [17], and they have significant implications for effective and accessible care.

In this large study, our primary finding is that compared to in-person visits, telemedicine was associated with 13% lower odds of missed appointments. This finding aligns with a pre-pandemic study from an immunology outpatient service line that reported 16% and 9% missed appointment rates for in-person and telemedicine services respectively [18]. This reduction associated with telemedicine is very encouraging because having fewer missed appointments translates to fewer disruptions in patient-clinician relationships [19], medication continuity, and can help close gaps in care. For patients, it can also translate to early or late disease detection [20], creating significant implications for health care expenditures. For traditionally underserved populations, fewer missed primary care appointments can improve health outcomes and prevent unnecessary emergency department visits and inpatient hospitalizations. Many blame missed appointments on transportation issues [21], for example, nonmetropolitan residents report greater difficulty accessing medical care because of problems related to travel [22]. For this reason, telemedicine may help reduce missed appointment rates in areas where residents have historically faced geographic barriers [23].

Because missed appointments represent lost revenue, the observed differential patterns across a range of demographic characteristics for telemedicine and in-person appointments are worth noting. For example, individuals with more than two visits in the preceding 15 months (December 2018 to February 2020) were more likely to miss in-person appointments but less likely to miss telemedicine appointments. This suggests that those who have frequent medical visits or those living with chronic diseases may be better candidates for telemedicine use. As we move to value-based payment contracts, reducing missed appointments by offering both telemedicine and in-person options becomes an increasingly urgent approach to increasing satisfaction, increasing quality, and lowering costs.

After adjusting for telemedicine use, the likelihood of missed appointments varied by race and ethnicity, with African American and Hispanic patients reporting 61% and 19% higher odds compared to Whites. This finding aligns with previous studies reporting that Hispanic [14] and African American [15] patients are more likely to miss appointments. This trend is concerning, suggesting that telemedicine is not an all-encompassing solution for missed appointments, and points to more pressing issues. For example, minority populations historically have a greater distrust of the health care system, as evidenced by the lower COVID-19 vaccine uptake in these populations [24]. In addition, the ratio of copays/deductibles to household income may be much higher in African American and Hispanic families compared to their White and Asian counterparts, contributing to cost-related delays in seeking care and missing health care appointments [25].

Perhaps the most striking finding is that insurance status was the strongest correlate of missed appointments. Previous evidence has suggested that individuals who miss their appointments are more likely to have Medicaid insurance and are more likely to be uninsured [6,14]. Our findings further support these earlier results. In our study, uninsured patients were almost two times more likely to miss appointments, and patients with Medicaid coverage had 75% higher odds of missed appointments when compared to privately insured patients. Notably, this may reflect poor access to broadband services, lower education levels, lower health literacy, or lower socioeconomic strata, which, although not captured in this study, may cause them to postpone seeking care. Uninsured persons may have jobs that do not allow them to take time off for medical appointments. The fact that the strongest missed appointment correlate was a type of insurance (which is mostly out of the patient’s immediate control) highlights that this is a complex issue influenced by patients, health care systems, and state policies.

Additionally, our findings exhibit an association between the service line and missed appointments. When compared to family practice, mental health was 20% more likely to experience missed appointments, and future studies should explore the reasons underlying this finding. Conversely, paediatrics, senior care, and obstetrics/gynecology were less likely to experience missed appointments. Importantly, this finding may be correlated with the differential uptake of telemedicine by service line. For example, although telemedicine has various applications in paediatric care, vaccinations cannot be delivered during telemedicine visits. For obstetrics/gynecology, physical exams necessitate face-to-face interactions, while postpartum visits were conducted largely via telemedicine during the pandemic. The trends in senior care align with previous findings that geriatric primary care practices see fewer missed appointments than general primary care practices [26].

Finally, our stratified regression model by appointment type yielded noteworthy results. Some groups, like Hispanic patients, Black patients, older adults, and those not on private insurance, were more likely to miss both telemedicine and face-to-face appointments. In contrast, those with mental health concerns were less likely to miss telemedicine appointments but more likely to miss in-person appointments. This highlights telemedicine’s important role in addressing the stigma associated with physically showing up for mental health appointments. This indicates that telemedicine can reduce barriers preventing some from accessing mental health services. Our findings also reveal the preferences for appointments based on the metropolitan residence: those living in metropolitan areas were less likely to miss telemedicine appointments but more likely to miss in-person appointments. We hypothesize that those living in metropolitan areas may prefer accessing health care through technology and may have more robust internet access. This finding is consistent with another telemedicine study, which found that compared to nonusers, telemedicine users are more likely to live in urban settings [23].

While these findings are encouraging, this study is not without limitations. Because we utilized data from an FQHC in Texas consisting of many community-based clinics, our findings may not be generalizable to other types of clinics or to clinics in other US states. It is important to note that while older adults aged 65+ years made up only 4.8% of our sample, FQHCs historically serve older adults at a slightly higher percentage of the patient population. In 2019, for instance, 9.6% of the FQHC patient population reported by HRSA was aged 65+ years [27]. While telemedicine implementation during the current pandemic has increased access to care for those who seek care from FQHCs, there are several challenges and barriers to virtual health that were not captured in this study. Thus, it is unclear whether these results will persist when COVID-19 transmission rates are lower. Finally, the study design establishes associations but does not allow for the determination of causal pathways between telemedicine use and missed appointments. Despite these limitations, findings from this large study suggest that the utilization of telemedicine services offers a potential method to reduce missed appointments for underserved populations.

In conclusion, the problem of missed appointments is prevalent in a variety of health care systems, and community-based clinics are not exempt. It is crucial that we understand the factors that drive missed appointments in order to design interventions for specific populations and ultimately improve access to care, particularly for underserved populations. The financial pressures that come from providing care to low-income populations necessitate that health care providers use their time efficiently, and reducing missed appointment rates through telemedicine enables providers to do just that.

## Supplementary Material

Supplemental Material

## Data Availability

Given the nature of this research, participants of this study did not agree for their data to be shared publicly, so supporting data are not available.
