# Supplementary material for: Transition to telemedicine and its impact on missed appointments in community-based clinics
Source: Ann Med. 2021 Dec 31;54(1):98–107. doi: 10.1080/07853890.2021.2019826 (PMC8725902; doi:10.1080/07853890.2021.2019826)
Supplement: Supplemental Material [file IANN_A_2019826_SM8934.docx]

**Supplemental Table 1**

**Mixed effect logistic regression model of the relationship between telemedicine and missed appointments in patients 0-18 years (n=112,711)**

| **Variables** |  |  | |  | | MV adjusted OR^b^ | | | | | |
| --- | --- | --- | --- | --- | --- | --- | --- | --- | --- | --- | --- |
|  |  | **OR** |  | | **95%CI** | | | |  |  | **P value** |
| **Appointment type** |  |  |  | |  | |  |  |  |  |  |
| Face-to-face appointment |  | Ref. |  | |  | |  |  |  |  |  |
| Telemedicine appointment |  | 0.74 |  | | 0.70 | | - | 0.78 |  | < | 0.001 |
| **Gender** |  |  |  | |  | |  |  |  |  |  |
| Male |  | Ref. |  | |  | |  |  |  |  |  |
| Female |  | 0.97 |  | | 0.93 | | - | 1.02 |  |  | 0.212 |
| **Ethnicity** |  |  |  | |  | |  |  |  |  |  |
| Non-Hispanic |  | Ref. |  | |  | |  |  |  |  |  |
| Hispanic |  | 1.22 |  | | 1.16 | | - | 1.28 |  | < | 0.001 |
| **Race** |  |  |  | |  | |  |  |  |  |  |
| White |  | Ref. |  | |  | |  |  |  |  |  |
| Black or African American |  | 1.74 |  | | 1.62 | | - | 1.85 |  | < | 0.001 |
| Asian |  | 0.88 |  | | 0.74 | | - | 1.04 |  |  | 0.122 |
| American Indian, Alaska Native, Other Pacific Islander |  | 1.41 |  | | 1.16 | |  | 1.72 |  |  | 0.001 |
| Mixed Race |  | 1.27 |  | | 1.13 | | - | 1.43 |  | < | 0.001 |
| **Insurance Coverage** |  |  |  | |  | |  |  |  |  |  |
| Private Insurance |  | Ref. |  | |  | |  |  |  |  |  |
| Medicare |  | 2.06 |  | | 0.47 | | - | 9.06 |  |  | 0.338 |
| Medicaid |  | 1.70 |  | | 1.59 | | - | 1.82 |  | < | 0.001 |
| Uninsured |  | 1.88 |  | | 1.72 | | - | 2.06 |  | < | 0.001 |
| **Service Line** |  |  |  | |  | |  |  |  |  |  |
| Family Practice |  | Ref |  | |  | |  |  |  |  |  |
| Mental Health |  | 0.90 |  | | 0.79 | | - | 1.03 |  |  | 0.113 |
| Obstetrics & Gynecology |  | 0.87 |  | | 0.69 | | - | 1.09 |  |  | 0.213 |
| Pediatrics |  | 0.73 |  | | 0.65 | | - | 0.83 |  | < | 0.001 |
| Senior Care |  | 1.02 |  | | 0.08 | | - | 12.44 |  |  | 0.988 |
| **Metropolitan Status** |  |  |  | |  | |  |  |  |  |  |
| Non-metropolitan |  | Ref. |  | |  | |  |  |  |  |  |
| Metropolitan |  | 1.01 |  | | 0.87 | | - | 1.16 |  |  | 0.906 |
| **Distance from Clinic** |  |  |  | |  | |  |  |  |  |  |
| <5 miles |  | Ref. |  | |  | |  |  |  |  |  |
| 5-10 miles |  | 0.97 |  | | 0.93 | | - | 0.99 |  |  | 0.015 |
| 10-20 miles |  | 0.97 |  | | 0.92 | | - | 0.99 |  |  | 0.025 |
| 20-50 miles |  | 1.02 |  | | 0.95 | | - | 1.10 |  |  | 0.519 |
| ≥50 miles |  | 1.15 |  | | 0.96 | | - | 1.38 |  |  | 0.122 |
| **Medically Underserved Area (MUA) Status** |  |  |  | |  | |  |  |  |  |  |
| Non-MUA |  | Ref. |  | |  | |  |  |  |  |  |
| MUA |  | 0.98 |  | | 0.94 | | - | 1.02 |  |  | 0.303 |
| **Visit History (Dec 2018-Feb 2020)** |  |  |  | |  | |  |  |  |  |  |
| 1-2 visits |  | Ref. |  | |  | |  |  |  |  |  |
| 3-4 visits |  | 1.84 |  | | 1.73 | | - | 1.96 |  | < | 0.001 |
| 5+ visits |  | 1.67 |  | | 1.59 | | - | 1.76 |  | < | 0.001 |
| **Intraclass Correlation Coefficient for Random Effect** | | | | | | | | | | | |
|  |  | **ICC** |  | | **95% CI** | | | |  |  | **Std. Err.** |
| **Patient** |  | 0.20 |  | | 0.19 | | - | 0.22 |  |  | 0.006 |

**Supplemental Table 2**

**Mixed effect logistic regression model of the relationship between telemedicine and missed appointments in adult patients, 19 years and older (n=165,460)**

| **Variables** |  |  | |  | | MV adjusted OR^b^ | | | | | |
| --- | --- | --- | --- | --- | --- | --- | --- | --- | --- | --- | --- |
|  |  | **OR** |  | | **95%CI** | | | |  |  | **P value** |
| **Appointment type** |  |  |  | |  | |  |  |  |  |  |
| Face-to-face appointment |  | Ref. |  | |  | |  |  |  |  |  |
| Telemedicine appointment |  | 0.89 |  | | 0.85 | | - | 0.92 |  | < | 0.001 |
| **Gender** |  |  |  | |  | |  |  |  |  |  |
| Male |  | Ref. |  | |  | |  |  |  |  |  |
| Female |  | 1.08 |  | | 1.04 | | - | 1.13 |  | < | 0.001 |
| **Ethnicity** |  |  |  | |  | |  |  |  |  |  |
| Non-Hispanic |  | Ref. |  | |  | |  |  |  |  |  |
| Hispanic |  | 1.14 |  | | 1.09 | | - | 1.19 |  | < | 0.001 |
| **Race** |  |  |  | |  | |  |  |  |  |  |
| White |  | Ref. |  | |  | |  |  |  |  |  |
| Black or African American |  | 1.53 |  | | 1.44 | | - | 1.63 |  | < | 0.001 |
| Asian |  | 0.75 |  | | 0.67 | | - | 0.84 |  | < | 0.001 |
| American Indian, Alaska Native, Other Pacific Islander |  | 1.08 |  | | 0.92 | |  | 1.27 |  |  | 0.346 |
| Mixed Race |  | 1.25 |  | | 1.06 | | - | 1.46 |  |  | 0.006 |
| **Insurance Coverage** |  |  |  | |  | |  |  |  |  |  |
| Private Insurance |  | Ref. |  | |  | |  |  |  |  |  |
| Medicare |  | 1.28 |  | | 1.19 | | - | 1.38 |  | < | 0.001 |
| Medicaid |  | 1.70 |  | | 1.60 | | - | 1.80 |  | < | 0.001 |
| Uninsured |  | 1.90 |  | | 1.80 | | - | 1.99 |  | < | 0.001 |
| **Service Line** |  |  |  | |  | |  |  |  |  |  |
| Family Practice |  | Ref |  | |  | |  |  |  |  |  |
| Mental Health |  | 1.21 |  | | 1.15 | | - | 1.26 |  | < | 0.001 |
| Obstetrics & Gynecology |  | 0.95 |  | | 0.90 | | - | 0.99 |  |  | 0.017 |
| Pediatrics |  | 1.15 |  | | 0.88 | | - | 1.50 |  |  | 0.296 |
| Senior Care |  | 0.66 |  | | 0.60 | | - | 0.73 |  | < | 0.001 |
| **Metropolitan Status** |  |  |  | |  | |  |  |  |  |  |
| Non-metropolitan |  | Ref. |  | |  | |  |  |  |  |  |
| Metropolitan |  | 1.06 |  | | 0.96 | | - | 1.16 |  |  | 0.269 |
| **Distance from Clinic** |  |  |  | |  | |  |  |  |  |  |
| <5 miles |  | Ref. |  | |  | |  |  |  |  |  |
| 5-10 miles |  | 1.01 |  | | 0.93 | | - | 1.03 |  |  | 0.409 |
| 10-20 miles |  | 1.02 |  | | 0.94 | | - | 1.04 |  |  | 0.619 |
| 20-50 miles |  | 1.01 |  | | 0.92 | | - | 1.03 |  |  | 0.330 |
| ≥50 miles |  | 1.02 |  | | 0.94 | | - | 1.07 |  |  | 0.347 |
| **Medically Underserved Area (MUA) Status** |  |  |  | |  | |  |  |  |  |  |
| Non-MUA |  | Ref. |  | |  | |  |  |  |  |  |
| MUA |  | 0.93 |  | | 0.88 | | - | 0.95 |  | < | 0.001 |
| **Visit History (Dec 2018-Feb 2020)** |  |  |  | |  | |  |  |  |  |  |
| 1-2 visits |  | Ref. |  | |  | |  |  |  |  |  |
| 3-4 visits |  | 1.55 |  | | 1.47 | | - | 1.64 |  | < | 0.001 |
| 5+ visits |  | 1.58 |  | | 1.52 | | - | 1.65 |  | < | 0.001 |
| **Intraclass Correlation Coefficient for Random Effect** | | | | | | | | | | | |
|  |  | **ICC** |  | | **95% CI** | | | |  |  | **Std. Err.** |
| **Patient** |  | 0.20 |  | | 0.19 | | - | 0.21 |  |  | 0.005 |
